# Prevalence, species identification and genotyping *Cryptosporidium* from livestock and deer in a catchment in the Cairngorms with a history of a contaminated public water supply

**DOI:** 10.1186/s13071-015-0684-x

**Published:** 2015-02-04

**Authors:** Beth Wells, Hannah Shaw, Emily Hotchkiss, Janice Gilray, Remedios Ayton, James Green, Frank Katzer, Andrew Wells, Elisabeth Innes

**Affiliations:** Moredun Research Institute, Pentlands Science Park, Penicuik, Midlothian EH26 0PZ UK; Scottish Water, Juniper House, Heriot Watt Research Centre, Edinburgh, EH14 4AP UK; The Crown Estate, 6 Bells Brae, Edinburgh, EH4 3BJ UK

**Keywords:** *Cryptosporidium*, Livestock, Deer, Water, Catchment, *C. parvum*, Genotyping, Prevalence, Transmission

## Abstract

**Background:**

The apicomplexan parasite *Cryptosporidium* represents a threat to water quality and public health. An important zoonotic species involved in human cryptosporidiosis from contaminated water is *Cryptosporidium parvum* (*C. parvum*), the main reservoirs of which are known to be farm livestock particularly neonatal calves, although adult cattle, sheep, lambs and wildlife are also known to contribute to catchment loading of *C. parvum*. This study aimed to establish *Cryptosporidium* prevalence, species and genotype in livestock, deer and water in a catchment with a history of *Cryptosporidium* contamination in the public water supply.

**Methods:**

A novel method of processing adult ruminant faecal sample was used to concentrate oocysts, followed by a nested species specific multiplex (nssm) PCR, targeting the 18S rRNA gene, to speciate *Cryptosporidium*. A multilocus fragment typing (MLFT) tool was used, in addition to GP60 sequencing, to genotype *C. parvum* positive samples.

**Results:**

A very high prevalence of *Cryptosporidium* was detected, with speciation identifying a predominance of *C. parvum* in livestock, deer and water samples*.* Four GP60 subtypes were detected within *C. parvum* with the majority IIaA15G2R1 which was detected in all host species and on all farms. Multilocus fragment typing further differentiated these into 6 highly related multilocus genotypes.

**Conclusion:**

The high prevalence of *Cryptosporidium* detected was possibly due to a combination of the newly developed sample processing technique used and a reflection of the high rates of the parasite present in this catchment. The predominance of *C. parvum* in livestock and deer sampled in this study suggested that they represented a significant risk to water quality and public health. Genotyping results suggested that the parasite is being transmitted locally within the study area, possibly via free-roaming sheep and deer. Further studies are needed to verify particular host associations with subtypes/MLGs. Land and livestock management solutions to reduce *Cryptosporidium* on farm and in the catchment are planned with the aim to improve animal health and production as well as water quality and public health.

## Background

*Cryptosporidium* are environmentally ubiquitous protozoan parasites, some species of which, for example *C. parvum*, are zoonotic and can cause gastro-intestinal disease in neonatal livestock and susceptible humans. *C. parvum* is commonly associated with diarrhoea in susceptible hosts causing illness and even death, particularly in neonatal calves [1]. Normally disease is self-limiting but the host may shed huge numbers of oocysts causing the infection to spread rapidly in calving areas and into the environment where they can remain infective for several years depending on environmental conditions. Livestock are well known as the main reservoirs for *C. parvum* [2] which is epidemiologically associated with zoonotic transmission [3] and are the species responsible for up to 50% of human cryptosporidiosis cases [4]. Infected neonatal calves tend to shed high concentrations of *C. parvum* oocysts [5] and in postcode sectors in Scotland which have a higher ratio of farms to humans, an increased rate of *C. parvum* infection in humans has been recorded [6].

Water is considered an important mechanism in the transmission of *Cryptosporidium* as the oocysts are extremely tough and survive well in ambient temperatures and damp environments [7]. In addition, Scottish livestock pasture frequently surrounds catchment areas collecting water ultimately destined for drinking water. This causes problems for water providers as contamination of the supply with *Cryptosporidium* requires them to condemn supplies, issue public notices to boil water before drinking from the affected supply and provide alternative drinking water, usually in the form of bottled water (Scottish Water, Pers. Comm.). Due to increasing outbreaks of cryptosporidiosis, the Scottish Water Directive (2003) was introduced to legislate for routine sampling of all public water supplies depending on its *Cryptosporidium* risk. This was calculated using risk assessments and subsequent weightings for parameters which affect *Cryptosporidium* levels for individual supplies. One of the highest weightings was given to the presence of livestock in the catchment, and the weighting score doubles if there are calves or lambs present, or if grazing densities are high (http://www.scotland.gov.uk/Resource/Doc/26487/0013541.pdf). The risk weighting is increased if the stock has direct access to the water course and reduced if the catchment is fenced off. Deer are also considered to represent a zoonotic risk to water supplies but have a lower weighting than livestock reflecting the generally lower grazing densities.

As livestock are considered to be the main reservoirs of *Cryptosporidium* oocysts, it is critical to have accurate information on prevalence and the species of *Cryptosporidium* present in order to assess the risk to public health from zoonotic transmission of *Cryptosporidium* through drinking water. However, reports on the prevalence of *Cryptosporidium* and in particular *C. parvum,* in livestock and wildlife are highly variable [5,8-10] and, although wildlife have been reported to contribute to *Cryptosporidium* loading in surface waters [11] there is a lack of data relating to *Cryptosporidium* prevalence in wildlife species. One study, however, recently completed in a catchment in Cumbria did include samples from both livestock and wildlife (including roe deer, badger, fox, rabbit and pheasant). *C. parvum* was isolated from water samples and from calf, lamb, adult sheep and fox samples and it was concluded that the distribution of C*ryptosporidium* species in surface waters, livestock and wildlife were linked [12].

Assignment to species level is useful in determining zoonotic potential of the parasite. However, to determine transmission dynamics and source of infection, more discriminatory power is required [11]. Genotyping within the species *C. parvum* has previously been based on single locus sequencing of a polymorphic region of the GP60 gene, which has a putative role in virulence. Whilst this is a useful library typing tool, it does not provide adequate differentiation for local or regional epidemiological questions, such as outbreak investigations. A recent paper reviewed multilocus genotyping schemes for *C. parvum* in the literature, which are usually based on micro/mini-satellite regions [13]. In multilocus fragment typing (MLFT), repeat units within the genome (micro/mini-satellites) are amplified and length polymorphisms due to variable numbers of repeat motifs are the basis for genotyping. Alleles at different loci, or markers, are combined to give a multilocus genotype (MLG). Robinson and Chalmers [13] appear to favour this approach, due to the potential to provide rapid, cost-effective results that are discriminatory enough to address source attribution. However, currently no coordinated scheme has been widely adopted or fully validated, although promising results have been obtained in bovine-derived *C. parvum*, and work is ongoing to develop a consensus approach (Hotchkiss E, Gilray J, Brennan M, Christley R, Morrison L, Jonsson N, Innes EA and Katzer F: Development of a framework for genotyping bovine-derived *Cryptosporidium parvum*, using a multilocus fragment typing tool; submitted). The catchment featured in the current study has a historical record of *Cryptosporidium* contamination in the public water supply which has resulted in continuing costly intervention by Scottish Water in terms of installation of suitable filtration, frequent sampling and dealing with alternative supplies of drinking water during water supply contamination events. To identify contributing livestock and wildlife species to the catchment loading of *Cryptosporidium*, and *C. parvum* in particular, we investigated the prevalence of *Cryptosporidium* in livestock on 4 farms and wild red and roe deer populations in this catchment. *Cryptosporidium* positive samples were speciated, following which C*. parvum* positive samples were genotyped to clarify the potential source of water contamination and transmission within the catchment.

## Methods

### Livestock, deer and water sampling

**Figure 1:**
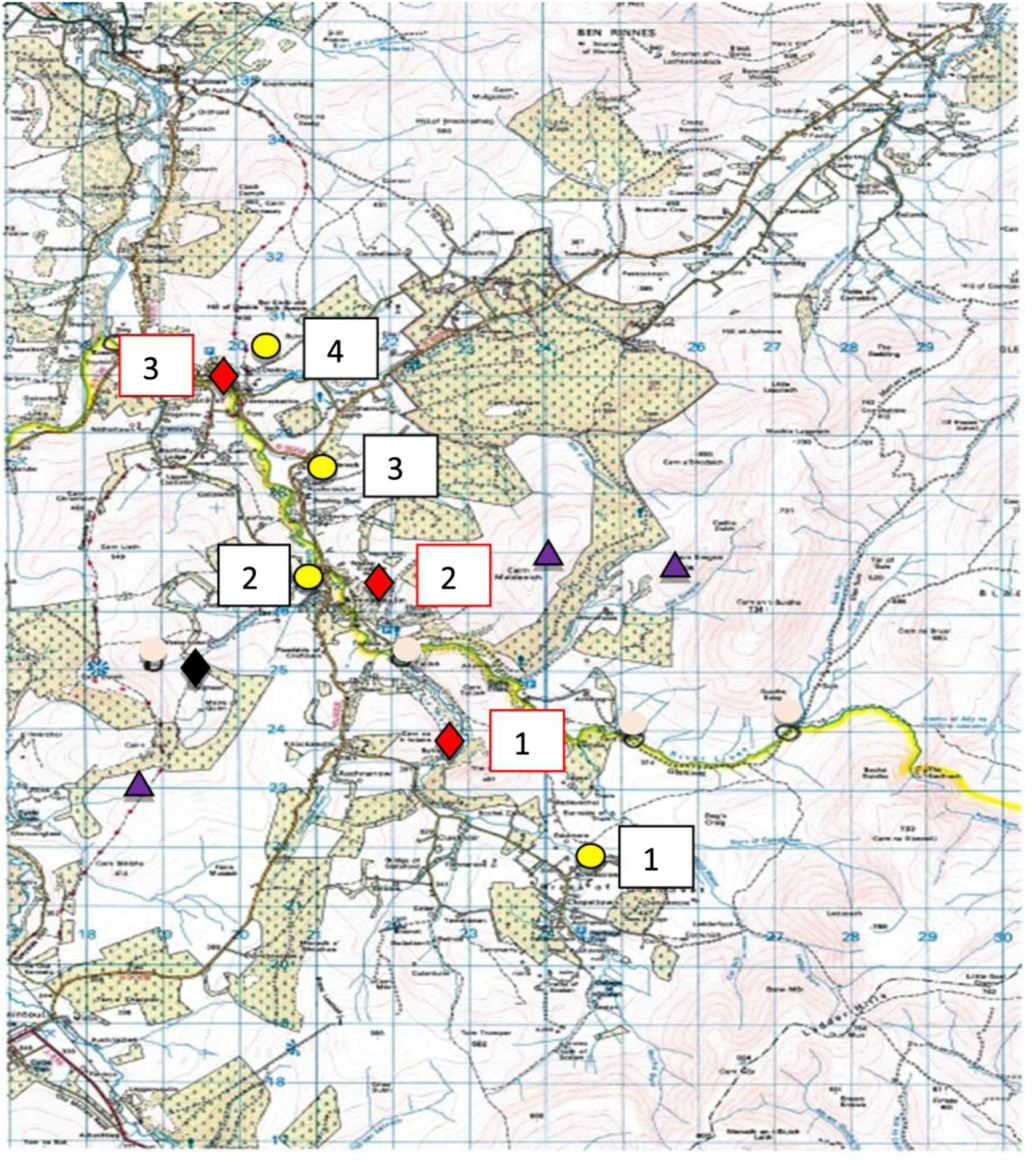
**Map of the catchment area sampling sites (Ordnance Survey Reference NJ22; Scale 1:55000).** “red diamond symbol” Water sampling sites (1–3); “black diamond symbol” Scottish Water public supply; “yellow circle symbol” Farms for livestock sampling (1–4) and “violet triangle symbol” Deer sampling areas. ©Crown copyright and database rights (2014) Ordnance Survey.

Samples were collected over 3 time points: late March, the first week in May and the first week in June. Four farms were selected for the study due to their location above, near and below the Scottish Water public supply intake (see Figure 1). The farms were all upland mixed livestock enterprises comprising medium sized beef herds and sheep flocks (Table 1).

**Table 1:** **Breeding cattle and sheep numbers (approximate) for the 4 study farms**

**Farm number**	**Herd numbers**	**Flock numbers**
1	80	400
2	150	2000
3	160	580
4	180	400

The roe and red deer were sampled in the approximate areas marked on Figure 1 according to deer sightings by gamekeepers and farmers. Water sampling sites (3 in total) were selected to allow representative sampling of the catchment.

Formal statistical measures of prevalence were not possible as samples were selected from different hosts, farms and at different sampling points based on availability of samples and practical issues with this type of field work.

#### Livestock samples

Faecal samples were collected from adult cattle, calves, sheep and lambs. During the first 2 sampling time points, cows and calves were housed and sampling was achieved by observation of the groups and collection of fresh faecal material ensuring sampled animals could be individually identified. Sheep and lambs were at pasture throughout the collection period and were also observed so that fresh, individual samples could be collected. A total of 157 livestock faecal samples were collected over the 3 sampling time points and the numbers sampled from each farm are shown in Table 2.

**Table 2:** **Total livestock numbers sampled on individual farms over the 3 sampling time points**

**Numbers sampled**	**Farm 1**	**Farm 2**	**Farm 3**	**Farm 4**	**Total no sampled**
Cows	4	10	7	9	30
Calves	8	14	16	19	57
Sheep	6	18	11	12	47
Lambs	9	6	3	5	23

#### Deer samples

Deer faecal samples were collected from the ground but unlike the livestock samples, were not necessarily fresh. This was the case for roe deer in particular as they are solitary, secretive animals and samples were found with difficulty. Six roe deer samples were collected from the catchment directly above the Scottish Water public supply intake for the village of Tomnavoulin. Twenty red deer faecal samples were collected from silage fields at grid reference (GR) 245 262 (Ordnance Survey Reference NJ22) from a herd of deer resident in the hills at the head of the catchment (Figure 1).

#### Water samples

Collection of water was performed according to standard operating protocols (SOPs) by the *Cryptosporidium* Laboratory, Scottish Water (SW) (http://www.scottishwater.co.uk). The 3 water sampling sites marked on Figure 1 (Site 1 = Braes of Glenlivet: OS map GR 226 234; Site 2 = Tomnavoulin: OS GR 213 261 and Site 3 = Glenlivet: OS GR 299 199) were sampled at each time point and the water volumes filtered are shown in Table 3. At the second time point, high water levels due to heavy rainfall meant that the pumps could not be used therefore 10 L grab samples were taken.

**Table 3:** **Water sample volumes filtered at each sampling site at each time point**

**Date of sampling**	**Sampling site**	**Volume filtered (L)**
27.03.14	1	182.3
	2	1007.5
	3	153.3
05.05.14	1	10 (grab)
	2	10 (grab)
	3	10 (grab)
03.06.14	1	125
	2	800
	3	219

### Sample processing and DNA extraction

#### Screening microscopy on livestock and deer samples

As a PCR check approximately 25% (n = 40) of livestock and deer samples, including those from all species and ages of animal and from all farms, were screened using light microscopy for the presence or absence of *Cryptosporidium* oocysts. Briefly, depending on the method of faecal sample processing as described in 2.2.2, 1 ml of either faecal suspension or suspension from the salt flotation pellet was added to a bijoux weighed and diluted 1:5 with dH_2_O. The sample was then vortexed vigorously and 100 μl added to 900 μl malachite green stain (0.16% malachite green, 1% SDS). Using a haemocytometer 10 μl of the stained faecal suspension was examined under the microscope for the presence of oocysts.

#### Livestock and deer

All adult cow, deer and sheep samples were processed by the most sensitive method available for concentrating oocysts in adult ruminant faecal samples (Wells B, Thomson S, Innes EA and Katzer F: Development of a sensitive method to extract and detect low numbers of *Cryptosporidium* oocysts from adult ruminant faecal samples; submitted). Briefly, 50 g of faeces was subjected to acid flocculation followed by salt flotation using the whole pellet obtained. The sample was then suspended in 1 ml TE buffer (10 mM Tris–HCl, 0.5 mM EDTA) mixed vigorously then centrifuged at 5,000 × g for 10 mins. The pellet was resuspended in 200 μl lysis buffer (T1 buffer, Macherey-Nagel, NZ740952250) and 10 freeze-thaw cycles in liquid nitrogen and a water bath at 56°C were performed. DNA was extracted using NucleoSpin Tissue DNA, RNA and Protein Purification Kits (Macherey-Nagel, NZ740952250) following the manufacturer’s protocol with the following modifications: the samples were incubated with Proteinase K at 56°C overnight following which the samples were vortexed vigorously and an additional incubation was performed at 95°C for 10 mins for the water samples only. Prior to the addition of ethanol, the samples were centrifuged at 11,000 × g for 5 mins to remove insoluble particles and the supernatant retained. Ultrapure water (100 μl) was used to elute DNA.

For lamb and calf samples where the animals were less than 1 month old for lambs and less than 3 months old for calves, samples were not processed prior to DNA extraction. Instead 250 μg (or 250 μl if liquid) of sample was added to 1 ml TE buffer. The protocol for adult samples described above was then followed. For older lambs (>1 month old) and calves (>3 months old) salt flotation using 3 g of faeces was performed prior to DNA extraction as this was found to improve oocyst concentration.

#### Water

Processing of filters, immunomagnetic separation (IMS) and microscopy were performed according to standard operating protocols (SOPs) by the *Cryptosporidium* Laboratory, SW (http://www.scottishwater.co.uk). Oocysts were identified microscopically using fluorescein isothiocyanate (FITC)–anti-*Cryptosporidium* monoclonal antibody (MAb) (FITC–*C*-MAb) and the nuclear fluorogen 4_,6-diamidino-2-phe-nylindole (DAPI) according to the Drinking Water Quality Regulator for Scotland (DWQRS) Standard Operating Protocol for Monitoring of *Cryptosporidium* Oocysts in Treated Water Supplies (http://www.dwqr.org.uk/technical/information-letters/public-2010). Slides with identified *Cryptosporidium* oocysts were collected from Scottish Water and the oocysts removed by adding 12 μl lysis buffer into the slide well and scraping the well with a loop. The liquid was then aspirated from the well into a tube containing 200 μl lysis buffer and the method followed as described in 2.2.2, with the additional step of two elutions using 50 μl ultrapure (UP) H_2_O followed by 25 μl UP H_2_O to maximise DNA yield.

### Polymerase Chain Reaction (PCR)

Amplification of DNA was by nested species specific multiplex PCR (nssm-PCR) targeting the 18S gene (Thomson S, Innes EA, Jonsson NN and Katzer F: A multiplex PCR test to identify four common cattle adapted *Cryptosporidium* species; submitted). Briefly, each 25 μl reaction contained 10× PCR buffer (45 mM Tris–HCl pH 8.8, 11 mM (NH4)2SO4, 4.5 mM MgCl2, 4.4 μM EDTA, 113 μg ml-1 BSA, 1 mM each of four deoxyribonucleotide triphosphates), 0.5 units BioTaq (Bioline, UK) and 10 μM of each primer. Primers for the common cattle species used were *C. parvum, C. andersoni, C. ryanae* and *C. bovis*. Other primer sequences used to amplify the common deer species *C. ubiquitum*, sheep species *C. xiaoi* and the common environmental species *C. suis* are shown in Table 4. DNA (3 μl) was added in the primary round and 1 μl primary PCR product in the secondary round for calf, adult cattle, sheep and lamb samples whereas the PCR was optimised for deer using 5 μl (first round) and 4 μl (second round) . The total volume was made up to 25 μl with dH_2_O. All reactions were carried out in triplicate and a positive; DNA extraction and negative control (dH_2_O) were included on each plate. Cycling conditions were 3 minutes at 94°C, followed by 35 cycles of 45 seconds at 94°C, 45 seconds at 55°C and 1 minute at 72°C. The final extension was 7 minutes at 72°C. Secondary amplification products (3 μl) were visualised on an AlphaImager 2000, following electrophoresis on a 1.5% Agarose gel stained with GelRed™ (Biotium, UK).

**Table 4:** **Additional primer sequences for primers used in the 18S nssm-PCR**

***Cryptosporidium*** **species**	**Primer sequence**	**Primer length**
*C. ubiquitum*	CAAGAAATAACAATACAGGACTTAAA	26
*C. xiaoi*	TTCTAAGAAAGAATAATGATTAATAGGA	28
*C. suis*	AAAGTTGTTGCAGTTAAAAAGCTT	24

### Sequencing

To confirm the nssm-PCR results, all positive water and deer samples were sent for Sanger sequencing (MWG Operon) along with a selection of samples from cattle, calves, sheep and lambs from each farm. The sequencing results were aligned with reference 18S rRNA sequences (GenBank, NCBI) for each possible *Cryptosporidium* species using BioEdit software [14].

### Genotyping

Six markers (MM5, MM18, MM19, TP14, MS1 and MS9) were used in a MLFT scheme which has been shown to perform well in calf *C. parvum* samples, in terms of typeability, specificity, repeatability and discriminatory ability (Hotchkiss E, Gilray J, Brennan M, Christley R, Morrison L, Jonsson N, Innes EA and Katzer F: Development of a framework for genotyping bovine-derived *Cryptosporidium parvum*, using a multilocus fragment typing tool; submitted). Nested PCR was carried out as described in Hotchkiss et al., with one second round primer fluorescently labelled; the resulting amplicons were sized by capillary electrophoresis via ABI 3730 (Applied Biosystems; University of Dundee), using size standard Genescan ROX500 (Applied Biosystems). In addition, a region of the GP60 gene was amplified [15] and sequenced to assign GP60 subtype, which was added to the allelic profiles of the 6 MLFT markers to assign MLGs. Only the primary peak was used in analysis where there was evidence of mixed alleles at one or more markers within a sample. Minimum spanning tree was created using PHYLOVIZ [16].

## Results

### All livestock

**Figure 2:**
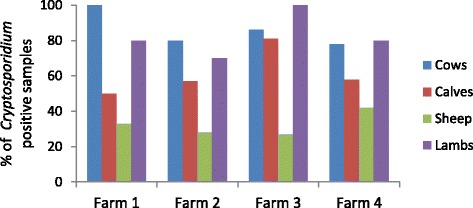
**Percentages of** ***Cryptosporidium*** **positive samples on each farm over all time points for each livestock species.**

Screening microscopy was performed on selected samples (n = 40) as described in section 2.2.1. Of those 40 samples, 23 (57%) were positive and 17 (43%) negative and all (100%) microscopy results agreed with the subsequent PCR results obtained. The study was completed over 3 sampling time periods which when compared, sample period 2 showed the highest prevalence of *Cryptosporidium* infection, calculated as an average of all 4 farms as shown in Table 5. A total of 157 livestock faecal samples were collected and analysed and the total percentages of *Cryptosporidium* positive samples for all farms over all time points are shown in Figure 2.

**Table 5:** **Percentages of the different livestock which tested positive for** ***Cryptosporidium*** **at each time point (total numbers tested in brackets)**

**Sampling period**	**Cattle**	**Calves**	**Sheep**	**Lambs**	**Mean of all livestock**
1	91 (n = 11)	33 (n = 27)	26 (n = 27)	n = 0	50 (n = 66)
2	80 (n = 15)	90 (n = 30)	31 (n = 16)	86 (n = 14)	73 (n = 79)
3	50 (n = 4)	n = 0	50 (n = 4)	67 (n = 9)	58 (n = 13)

#### Adult cattle

**Figure 3:**
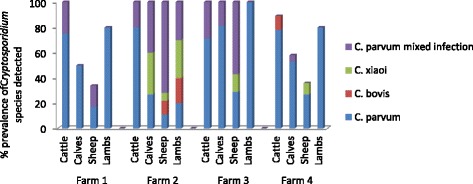
***Cryptosporidium*** **species prevalence in all farm livestock species in each farm for all time points.**

The adult cattle sampled had a consistently high prevalence of *Cryptosporidium* over all four farms and at all sampling points (Table 5 and Figure 2). *C. parvum* was the predominant species in adult cattle in all the farms (Table 6 and Figure 3) and there were only 2 mixed infections - one mixed *C. andersoni/C. parvum* infection and one mixed *C. bovis/C. parvum* infection.

**Table 6:** **Prevalence of C. parvum in livestock in all farms over all time points, as a percentage of the** ***Cryptosporidium*** **positive samples detected**

**Livestock**	***C. parvum*** **prevalence as % of** ***Cryptosporidium*** **positive samples**
Cattle	96
Calves	100
Sheep	71
Lambs	89

#### Calves

Farms 1, 2 and 4 showed similar *Cryptosporidium* prevalence in calves ranging from 50 to 58% (see Figure 2) with Farm 3 showing a much higher prevalence (81%). At the first sampling point, the *Cryptosporidium* prevalence averaged over all four farms was low (33%) but by the second sampling point, this had increased to 90%. As shown in Figure 3, *Cryptosporidium* positive calf samples were determined to be *C. parvum* by nssm-PCR, with only 5 calves showing mixed infections, 3 of these being *C. parvum/ C. bovis,* 1 *C. parvum/C. ryanae* and 1 *C. parvum/ C. bovis/ C. ryanae.*

#### Sheep

The sheep samples yielded the lowest incidence of *Cryptosporidium* infection on all farms compared to the adult cattle and deer samples, ranging from 27 – 42% (Figure 2). All farms had a similar prevalence over the time points sampled. Of the 14 samples analysed from sheep which tested positive for *Cryptosporidium*, there were 8 *C. parvum* infections; 4 *C. xiaoi* infections and 2 mixed infections – 1 *C. parvum/C. xiaoi* and the other *C. parvum/C. xiaoi/C. ubiquitum* (see Figure 3)*.*

#### Lambs

*Cryptosporidium* infection in lambs sampled was at a high prevalence of 78%. There were no lambs born at the first sampling point and the incidence of *Cryptosporidium* infection was higher at the 2^nd^ sampling point (86%) compared to the 3^rd^ (67%) (Table 5). The PCR results indicated that 13 of the *Cryptosporidium* positive samples were *C. parvum*, 2 were *C. xiaoi* infections and 3 were mixed infections – 2 *C. parvum/ C. xiaoi* and 1 *C. parvum/C. ubiquitum*.

#### Prevalence of *C. parvum* in all livestock

The high prevalence of *C. parvum* in all livestock samples analysed in the 4 farms is shown in Figure 3 and Table 6. The *C. parvum* prevalence was highest in calves and as an average of all livestock it was 89%.

### Deer

#### Red deer

Of the 20 individual red deer samples analysed, 80% were positive for *Cryptosporidium* by PCR. On speciation by 18S nssm PCR, 87.5% of these *Cryptosporidium* positive deer had *C. parvum* infections (12.5% of which were mixed infections with *C*. deer genotype) and the remaining 12.5% had *C*. deer genotype infection.

#### Roe deer

Of the 6 roe deer samples collected, 2 were *C. parvum* positive by PCR, a total of 33%.

### Water

All water samples which were positive for *Cryptosporidium* oocysts by microscopy, had DNA extracted which amplified by 18S nssm PCR as *C. parvum* (3 samples in sampling periods 1 and 2) or *C. xiaoi* (1 sample in sampling period 3) see Table 7. All PCR amplicons (triplicate) from all the *Cryptosporidium* positive water samples were sequenced, the sequencing results confirming the PCR results.

**Table 7:** **Microscopy (SW) and 18S nssm-PCR results from water samples taken from the 3 sites sampled over 3 time periods**

**Date of sampling**	**Sampling site**	**Crypto oocyst Count/10 L**	**18S nssm PCR result**	**Species confirmed (sequenced)**
27.03.14	1	2	*C. parvum*	*C. parvum*
	2	<0.2	Neg	
	3	0.8	Neg	
05.05.14	1	1.67	*C. parvum*	*C. parvum*
	2	<0.2	Neg	
	3	2	*C. parvum*	*C. parvum*
03.06.14	1	0	Neg	
	2	0.3	*C. xiaoi*	*C. xiaoi*
	3	0	Neg	

### Sequencing

For all livestock, one *C. parvum* positive sample from each farm and several mixed infection samples were sequenced and the aligned sequences confirmed the PCR results apart from *C. ubiquitum* in the mixed infections in the sheep samples. These sequences once aligned identified highly with *C. parvum* and *C. xiaoi* but *C. ubiquitum* was not identified. All the lamb samples collected at time point 3 were sequenced as they were grazing on pasture directly above the Scottish Water public supply intake. The *Cryptosporidium* species isolated here were *C. parvum, C. xiaoi* and mixed infections of both.

All positive red deer samples were sequenced and all *C. parvum* and deer genotype positives were confirmed by the sequencing results. Species which were detected as *C. ryanae* by PCR aligned most closely to *C*. deer genotype using BioEdit. Both *C. parvum* positive samples by nssm 18S PCR from roe deer were sequenced but the resulting sequences were very poor quality, even on re-sequencing, suggesting poor quality DNA and therefore the PCR results could not be confirmed. All other confirmed sequences showed 98-100% identity with reference sequences (GenBank) and 7 were submitted to NCBI (accession numbers shown in Table 8).

**Table 8:** **GenBank (NCBI) accession numbers for a selection of the sequenced samples**

**Sample type**	***Cryptosporidium*** **species detected**	**Identity (%) to reference species**	**GenBank accession number**
Bovine - cow	*C. parvum*	99	KP004200
Ovine - ewe	*C. xiaoi*	99	KP004201
Bovine - calf	*C. parvum*	99	KP004202
Ovine - lamb	*C. xiaoi*	100	KP004203
Cervine - Hind	*C. parvum*	100	KP004204
Cervine - hind	C. deer genotype	99	KP004205
Water - raw	*C. parvum*	100	KP004206

### Genotyping using GP60 and Multi Locus Fragment Typing (MLFT)

#### GP60 subtyping

*C. parvum* and mixed species including *C. parvum* positive samples were analysed by GP60 PCR (n = 112). Sequencing the 89 positive results from this gave 66 readable traces. All calf samples (23/23) and the majority of cow samples (14/16) were IIaA15G2R1 on all farms (Table 9). This subtype was the most prevalent (53/66), being identified in all sample types. Six samples were IIaA19G2R1 of which 5 were from sheep or lambs from farms 2, 3 and 4; the other sample was from a deer. Subtype IIaA18G2R1 was also identified in 6 samples, 3 of which came from deer, with one lamb and one cow also shedding the subtype. This subtype was also detected in water (Table 9). In terms of farm of origin, the IIaA15G2R1 GP60 subtype was identified on each of the 4 farms, with IIaA18G2R1 being found on farms 2 and 4, IIaA19G2R on farms 2, 3 and 4 and finally IIaA14G2R1 on farm 2 only.

**Table 9:** **GP60 subtype of 66** ***Cryptosporidium parvum*** **samples, according to host of origin**

	**IIaA15G2R1**	**IIaA18G2R1**	**IIaA19G2R1**	**IIaA14G2R1**	**Total**
Calf	23	0	0	0	23
Cow	14	1	0	1	16
Sheep	1	0	3	0	4
Lamb	6	1	2	0	9
Deer	8	3	1	0	12
Water	1	1	0	0	2
Total	53	6	6	1	66

#### MLFT

**Figure 4:**
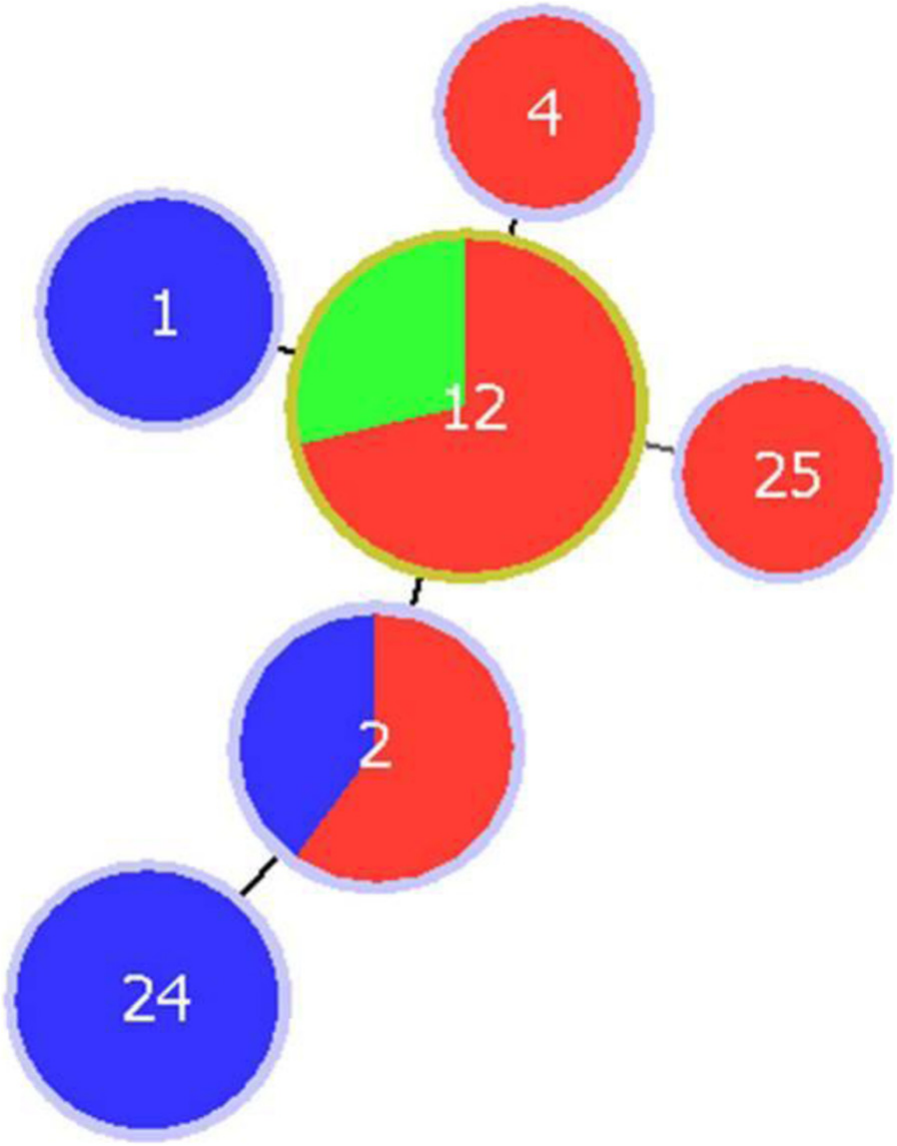
**Minimum spanning tree showing the relationships between 6 MLGs identified in 27** ***Cryptosporidium parvum*** **samples, according to host species.** Blue represents samples from adult cattle (n = 8), red represents calf samples (n = 15), green represents lamb samples (n = 4). Each circle represents a MLG and the diameter is proportional to the number of samples of that MLG. MLGs are joined by a line if they share 6 of 7 alleles.

Twenty seven samples were successfully typed at all 6 MLFT loci and GP60 locus, and included cow, calf and lamb samples representing the GP60 subtype IIaA15G2R1 only. The 6 MLGs detected all formed a clonal complex (with the criterion for clonal complex membership being sharing at least 6/7 alleles) indicating that they were highly related (Figure 4). Within farms, cows and calves had different MLGs; on farm 3 lambs and calves shared a MLG (Table 10).

**Table 10:** **Multilocus genotypes of** ***C. parvum*** **detected in 27 samples according to farm and host of origin**

	**MLG 1**	**MLG 2**	**MLG 4**	**MLG 12**	**MLG 24**	**MLG 25**	**Total**
Farm 1	1 (cow)	4 (calves)	0	0	0	0	5
Farm 2	0	0	0	2 (lambs)	1 (cow)	1 (calf)	4
Farm 3	1 (cow)	1 (cow)	1 (calf)	7 (calves & lambs)	0	0	10
Farm 4	0	0	0	5 (calves)	3 (cows)	0	8
Total	2 (cows)	5 (calves & cow)	1 (calf)	14 (calves & lambs)	4 (cows)	1 (calf)	27

## Discussion

The overall levels of *Cryptosporidium* isolated in this study indicated highest prevalence in early May when there were high numbers of neonatal livestock in the catchment. Cattle, and particularly young calves, are major reservoirs of *Cryptosporidium* [17,18] and have been associated with increased waterborne human infection risks [19]. In this catchment, historical data indicated that the highest levels of *Cryptosporidium* oocysts were found in mid-late summer and were associated with high intensity rainfall events (Scottish Water, Pers. Comm.) and this has also been associated with higher human infection risk from waterborne *Cryptosporidium* in other catchments [19]. In the study catchment, cows and calves remained housed until the end of May, so the later water infection period seen by Scottish Water may reflect the increased presence of cattle in the catchment, as well as the time of year with higher monthly rainfall totals (http://www.metoffice.gov.uk/climate/uk/datasets/Rainfall/ranked/Scotland_N.txt).

*Cryptosporidium* infection was highly prevalent in tested cattle, calves and lambs in the 4 farms sampled in this catchment, but lower in tested sheep. This was evident in all 4 farms in the study which showed consistent results across the farms sampled (Figure 2). Farm 3, which had the highest infection level in tested calves and lambs, had serious problems with cryptosporidiosis in calves born the previous autumn so it is likely that there were viable oocysts remaining in the calving sheds and the fields from previous infection cycles. All the new born calves on this farm were treated with halofuginone lactate (Halocur™, MSD Animal Health) at birth and for 7 consecutive days after. This had the effect of reducing the clinical signs seen previously but the treated calves continued to shed oocysts consistent with previous studies on the effect of halofuginone lactate on cryptosporidiosis in calves [20].

The prevalence of *Cryptosporidium* in tested cattle in this study was highest (91%) in late March which was early in the spring calving period, and averaged 80% in early May when the calving period was nearly finished. This prevalence in tested cattle is much higher than has previously been reported [5,8,9] and is likely to be, at least in part, due to the increased sensitivity of the method of concentrating *Cryptosporidium* oocysts in adult cattle samples (Wells B, Thomson S, Innes EA and Katzer F: Development of a sensitive method to extract and detect low numbers of *Cryptosporidium* oocysts from adult ruminant faecal samples; submitted). This method includes a combination of acid flocculation using 50 g of starting faecal material, combined with salt flotation, and resulted in increased *Cryptosporidium* detection from 4.78% to 29% in 209 samples from dairy cattle. However, this does not fully explain the higher prevalence of *Cryptosporidium* detected in the cattle in this study, where the samples were collected from individual cattle in the peri-parturient period. There is conflicting evidence for a peri-parturient rise in *Cryptosporidium* oocyst output by cattle [10,21] but it may be one reason for the very high prevalence seen in peri-parturient cattle in this study.

There was also a high prevalence of *Cryptosporidium* in the tested calves on all 4 farms. Calves are considered the main reservoirs for the parasite but even so, the levels found here were high compared with many studies [22,23]. There was an increase in *Cryptosporidium* prevalence between sampling point 1 and 2 in the calf samples, which suggested that as the calving progressed, the calf shed environment became increasingly contaminated with oocysts leading to higher rates of infection in newborn calves [24]. This was particularly evident on farm 4 where 2/7 calves were infected at time point 1 and 9/10 at time point 2. Between the 2 sampling dates this farm had a serious outbreak of cryptosporidiosis in the calves. No calves died but many were sick and required veterinary attention and rehydration therapy.

In contrast, although overall prevalence of *Cryptosporidium* infection in tested lambs was high (78%), it fell between sampling periods 2 (early lambing) and 3 (post lambing) (Table 5). During the early lambing sampling period at time point 2, most lambs were neonatal (0–1 month old) and more susceptible to infection compared to time point 3. In addition, grazing densities of ewes were high at time point 2 as all the ewes were held in lambing fields to allow ease of access for lambing. Although the prevalence of *Cryptosporidium* in sheep was lower than for cattle, sheep remain grazing on pasture in the catchment all year round and therefore are likely to contribute significantly to catchment loading of the parasite. PCR results for both sheep and lambs showed that *C. ubiquitum* was present in mixed infections but sequencing failed to confirm this, suggesting that either the *C. ubiquitum* primers used were not specific, or the other species in the mixed infections were preferentially amplified during PCR. *C. ubiquitum* was one of the zoonotic species responsible for water supply contamination in the catchment (Scottish Water, Pers. Comm.), so it was disappointing that we could not confirm its presence in sheep. Interestingly, *C. parvum* was the predominant species detected throughout May 2014 (Scottish Water, Pers. Comm.) which confirms the predominant species found in livestock and wildlife tested at this time.

Differences in *Cryptosporidium* prevalence between calves and adult cattle (and sheep and lambs) cannot be compared due to the different sample processing techniques used prior to DNA extraction. For example, adult cattle and sheep samples were processed using 50 g faeces compared to calves and lambs where 250 mg were used.

The most prevalent species of *Cryptosporidium* isolated during this study was *C. parvum* which was found in 89% of *Cryptosporidium* positive livestock samples. The high proportion of *C. parvum* found in all the livestock species has previously been reported in lambs and calves [25,26] but it is unusual to find such high *C. parvum* prevalence in adult cattle and sheep [5,27]. Approximately half of the estimated laboratory confirmed cases of cryptosporidiosis in the UK are estimated to be caused by *C. parvum* [4] therefore the high prevalence of *C. parvum* in all livestock types in this study suggest that they represent a significant risk to water quality and public health in this catchment.

**Figure 5:**
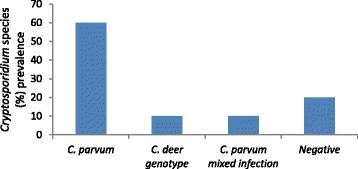
***Cryptosporidium*** **species prevalence in Glenlivet red deer (n = 20).**

In addition to investigating the levels of *Cryptosporidium* infection in livestock, this study aimed to assess the contribution of the wild deer population to *Cryptosporidium* burden in the catchment. The prevalence of *Cryptosporidium* detected in the Glenlivet red deer samples was 80% (Figure 5). A previous study which monitored levels of *Cryptosporidium* in farmed red deer hinds (n = 40) and calves sampled monthly over a 1 year period, reported asymptomatic low level shedding of oocysts all year with 39.3% of samples from hinds and 60% of samples from calves being positive for *Cryptosporidium* [28]. As our samples all came from adult wild red deer, the *Cryptosporidium* prevalence within the tested red deer population in Glenlivet is surprising. It is known that the red deer population in Glenlivet is increasing (The Crown Estate, Pers. Comm.) and the samples were collected from a fenced area where the farmer was hoping to crop silage, therefore grazing densities in this area would have been higher than normal for a wild deer herd, potentially leading to the higher infection rates.

The predominant *Cryptosporidium* species in red deer was *C. parvum,* with 70% of all deer samples being positive with either a single *C. parvum* infection (60%) or a mixed infection involving *C. parvum* (10%). Wildlife has previously been reported to contribute to parasite loading in catchments but at a lower order of magnitude than livestock [29]. It is likely that the relative amount of parasite harboured by the deer was low compared with livestock, reflected by the required re-optimisation of the 18 s rRNA PCR for detection of *Cryptosporidium* in the deer samples, using more DNA and PCR product (section 2.3). These results reflect the risk weightings contained in the Scottish Water Directive (2003) where deer have a lower weighting than adult livestock, which have a lower weighting than neonatal livestock (http://www.scotland.gov.uk/Resource/Doc/26487/0013541.pdf). However, due to the very high prevalence of *C. parvum* in red deer in this study, further investigation using real-time PCR, to allow quantification of DNA, is planned with all the *C. parvum* positive samples to estimate the relative contribution of parasite loading in the catchment from livestock and wildlife.

Roe deer samples (n = 6) showed lower *Cryptosporidium* prevalence ( 33%) but these were all speciated as *C. parvum* and therefore these deer are also likely to be of relevance when considering water quality and zoonotic transmission of *Cryptosporidium* in the catchment. The predominance of *C. parvum* is unusual in roe deer as previous reported studies had isolated only *C. ubiquitum* and *C.* deer genotype [12] or had not speciated but had identified a low (1.3% of 224 sampled) but widespread level of *Cryptosporidium* in roe deer in Galicia, NW Spain [30]. As with red deer, the numbers of roe deer sightings in this catchment have been reported as increasing (David Newland, Glenlivet Wildlife; Pers. Comm.) particularly around the corrie above the SW public water supply intake, which may increase the risk of contamination.

The species of *Cryptosporidium* isolated from the water sampling sites reflected the predominant species found in the livestock at that particular time, providing further evidence for direct transfer of oocysts from grazing animals into the catchment water systems. This has previously been recorded in surface water contamination with *C. parvum,* which was linked to *C. parvum* isolated from calves grazing near the water course [31]. Robinson *et al.* [12] also reported this link between the species of *Cryptosporidium* excreted by livestock and wildlife with that found in surface waters of the catchment. The historical data for *Cryptosporidium* isolated from the study catchment’s public water supply (Scottish Water, Unpublished Data) also supports the suggestion that the isolation of *C. parvum* in the public water supply is linked to the high prevalence of *C. parvum* in all the livestock species and deer tested in the catchment.

Sampling period 2 was preceded by heavy rainfall which resulted in smaller volume grab samples being taken compared to the volumes of samples filtered in sampling periods 1 and 3. Despite this difference in sampling volume, it is interesting that *Cryptosporidium* oocysts were recorded for 2/3 sites at sampling period 2 compared to 1/3 for sampling periods 1 and 3. This may reflect the effect of rainfall on oocyst concentration in surface water run-off, which has previously been considered as a significant factor in detecting oocysts in surface waters [12,32].

In an attempt to further investigate the relationships between potential sources of *C. parvum* oocysts in the catchment, genotyping was carried out using both the established library typing tool of GP60 sequencing and a more discriminatory method based on multilocus fragment typing [13]. As in previous studies in cattle, IIaA15G2R1 was the most prevalent GP60 subtype [15] and was shed by all host species. However it appears to be particularly associated with young calves, as no other subtype was detected in this group. Results suggested that subtype IIaA19G2R1 may be associated with ovine hosts and IIaA18G2R1 with cervine hosts, but this should be interpreted with caution due to the low numbers of samples subtyped in this study. Greater numbers of samples from different hosts should be typed to investigate this apparent host-association further as this may be an important indicator of source in outbreak investigations. The GP60 subtypes identified in water samples were those associated with livestock and deer in this study, suggesting that all of these have potentially a role to play in *C. parvum* contamination of the water sources.

MLFT differentiated the common GP60 subtype IIaA15G2R1 into 6 highly related MLGs, demonstrating the greater discriminatory power of this tool. However typeability was disappointing, with only 27 samples amplifying at all 6 MLFT markers and GP60. There may be several reasons for this. The MLFT was optimised for use in calves, which have been shown to shed *C. parvum* oocysts in great numbers. Adult ruminants are likely to have reduced concentration of oocysts in a greater volume of faecal output. Attempts were made to address this by pre-processing of these sample types as described in 2.2.2, to allow more of the original material to be processed. However, even with these measures, it is likely that MLFT PCRs should be further optimised for these sample types, as was done with 18S rRNA PCR (section 2.3). It would also be extremely beneficial to be able to genotype oocysts in water; however the low numbers typically obtained may preclude this, using current protocols.

There was a statistically significant difference in prevalence of mixed MLGs in cows (6/8) compared to detected in young calves and lambs (2/19) (Fisher’s exact test P = 0.002). This is likely to be due to the fact that adult animals will have had exposure to a greater number and variety of sources of oocysts. In addition, calves encountering the oocysts for the first time can become acutely infected with a low infectious dose which is massively amplified in the small intestine [33], resulting in clonal expansion of one MLG.

The fact that all 4 farms have very closely related MLGs is not surprising for several reasons. Firstly, it has been shown in previous studies using this typing tool that *C. parvum* is relatively conserved, certainly in bovine species (Hotchkiss E, Gilray J, Brennan M, Christley R, Morrison L, Jonsson N, Innes EA and Katzer F: Development of a framework for genotyping bovine-derived *Cryptosporidium parvum*, using a multilocus fragment typing tool; submitted) and the 4 farms in this study are very close geographically. In addition, certain farm management practices would allow transmission between farms as, for example, sheep are often co-grazed on the hill pastures and in this area, free-roaming deer graze in livestock pastures and have been shown in this study to be reservoirs of *C. parvum*.

At the farm level, the limited data available showed that adult cows had different MLGs to calves. This is important in elucidating transmission routes and epidemiology within-farm and suggests that dams may not be the source of infective oocysts for calves, although numbers are too low to be conclusive and paired calf-dam samples were not specifically sought. However this is an area of interest as it may influence advice on farm management control strategies which can be prioritised towards reducing calf-to-calf transmission, rather than dam to calf.

## Conclusions

In conclusion, the apparently higher prevalence of *Cryptosporidium,* and *C. parvum* in particular, detected in livestock and deer samples tested in this study compared to previous studies, along with the historical problems of *Cryptosporidium* infection in the public supply water source and other surface waters tested, suggests that transmission of the parasite through the catchment is cyclic with re-infection occurring on a seasonal basis linked to the ability of *Cryptosporidium* oocysts to survive in the environment. This high prevalence of *C. parvum* represents a threat to water quality and public health. In response to this, Scottish Water, as the public water provider and the Crown Estate as the landowner are planning the erection of catchment fencing and provision of water troughs away from the public water supply intake. However, as the catchment supplies not only a public water supply, but numerous private supplies which are not tested, the risk must be high in the untested water supplies. Discussions are ongoing and meetings planned to help inform farmers and land managers of management options available to reduce the prevalence of *Cryptosporidium* on farm, with benefits to animal health and production as well as water quality and public health.
